# Cationized Decalcified Bone Matrix for Infected Bone Defect Treatment

**DOI:** 10.34133/bmef.0066

**Published:** 2024-10-02

**Authors:** Le Chen, Yuying Ai, Ruonan Wu, Zhaoyan Guo, Yang Li, Jie Li, Feng Qu, Shun Duan, Fu-Jian Xu

**Affiliations:** ^1^ State Key Laboratory of Chemical Resource Engineering, Key Lab of Biomedical Materials of Natural Macromolecules ( Beijing University of Chemical Technology), Ministry of Education, Beijing Laboratory of Biomedical Materials, Beijing University of Chemical Technology, Beijing 100029, China.; ^2^ Beijing Research Institute of Chemical Industry, Sinopec, Beijing 100013, China.; ^3^ Beijing Chaoyang Hospital, Capital Medical University, 100020, China.

## Abstract

**Objective:** We aim to develop a dual-functional bone regeneration scaffold (Q*x*-D) with antibacterial and osteogenic properties for infected bone defect treatment. **Impact Statement:** This study provides insights into antibacterial components that could be combined with naturally derived materials through a facile Schiff base reaction, offering a potential strategy to enhance antibacterial properties. **Introduction:** Naturally derived decalcified bone matrix (DBM) has been reported to be porous and biodegradable. DBM can induce various cell differentiations and participate in immune regulation, making it an ideal bone regeneration scaffold for bone defects. However, DBM does not exhibit antimicrobial properties. Therefore, it is essential to develop antibacterial functionalization method for DBM. **Methods:** DBM was modified with a macromolecular quaternary ammonium salt (QPEI). A series of Q*x*-D with tunable feeding ratios were synthesized through Schiff base reaction. The morphology, chemical property, in vitro antibacterial efficiency, in vitro biocompatibility, osteogenic property, and in vivo anti-infection performances were characterized. **Results:** All Q*x*-D exhibited marked antibacterial properties. Small adjustments in feed concentration could not induce changes in antibacterial properties. However, cell viability slightly decreased with increasing feed concentration. Q10-D demonstrated significant antibacterial properties and could promote recovery of infected bone defect in an animal model. **Conclusion:** Q*x*-D shows marked antibacterial properties and good biocompatibility. Moreover, Q10-D could be a potential choice for infected bone defects.

## Introduction

A series of factors might lead to bone defects, such as tumor, trauma, necrosis, and congenital deformity. According to statistics, approximately 20 million patients worldwide suffered from bone defect due to various diseases every year [1]. Among them, the treatment of infected bone defects (IBDs) caused by severe trauma, improper early management, and surgical infection was still a very challenging problem in orthopedic clinic. The main pathogens of bone infection were Gram-positive bacteria, such as *Staphylococcus aureus* (*S. aureus*), methicillin-resistant *S. aureus* (MRSA), and coagulase-negative *Staphylococcus* (CoNS). [2,3] In addition, bones might also be infected with Gram-negative bacteria, such as *Escherichia coli* (*E. coli*) and *Pseudomonas aeruginosa* (*P. aeruginosa*) [4]. Bacteria attached to the surface of the bone could grow and form a biofilm. The biofilm acted as a barrier that could make it difficult for antimicrobials to penetrate, hindering the effect of antibiotics and other drugs on bacteria [5]. What is more, biofilms could promote the reproduction of bacteria, further complicating the eradication of bacteria and increasing the complexity and difficulty of treatment. Besides, bacteria would compete with cells for adhesion sites, impeding the growth of stem cells or osteoblasts. Severe bacterial infection could also lead to complications and implant failure. For example, the morbidity of bacterial infection in clinical implants exceeded 20% in the United States [6]. Therefore, the treatment of IBDs had important clinical and scientific significance.

The traditional standard treatment of IBDs included 2 main tasks, namely, infection control and bone defect reconstruction [7,8]. The first stage was debridement and fixation of the fracture site, and the second stage was treatment of infection and repair of bone defect. At present, naturally derived decalcified bone matrix (DBM) scaffolds had been reported to be porous and biodegradable, inducing various progenitor cells and macrophages to chemotaxis and participate in immune regulation [9]. It had been shown that DBM-based hydrogels could enhance the proliferation of osteoblasts [10]. In another study, DBM/alginate hydrogel with growth factor improved mineralization and bone tissue regeneration of femur defects [11]. Additionally, the surface of DBM contained a large number of active carboxyl groups and amino groups, which could be used to modify functional components to enhance their properties [12]. However, DBM exhibited little antibacterial properties. A plenty of strategies have been developed to construct antibacterial surfaces [13–18], such as antibiotic loading [19], host defense peptide mimicking [20], photothermal coating [21], photosensitizer modification [22], and polymer functionalization [23–25]. For example, Fang et al. [26] cross-linked vancomycin with specific acellular cancellous bone through electrostatic interaction and chemical bonding to construct a new antibacterial material for the treatment of multifunctional bone infection. However, because of the problems of drug resistance and the bioactivity of DBM, a novel strategy for antibacterial functionalization of DBM still remains a challenge.

Cationic polymer quaternary ammonium salt (QPEI), antibacterial components, has been used to construct dual-functional implants with antibacterial and osteointegration-promoting performances [27], dual-modal drug-loaded nano-assemblies to treat multidrug-resistant bacterium-induced lower respiratory tract infections [13], and many other biomedical materials, but there was little cytotoxicity. Because of its strong antibacterial properties, QPEI is widely used in various sterilization and disinfection applications. Studies have shown that QPEI has a good inhibitory effect on a variety of bacteria, fungi, and viruses [28,29]. QPEI can be chemically modified or introduced into a sustained release system to reduce the side effects on the human body. What is more, QPEI can be modified with functional groups to improve its stability, antibacterial effect, and biocompatibility. QPEI can also be compounded with other materials to develop new functional materials, such as antibacterial textiles and antibacterial coatings. These provide a feasible way for further transformation.

**Fig. 1.**
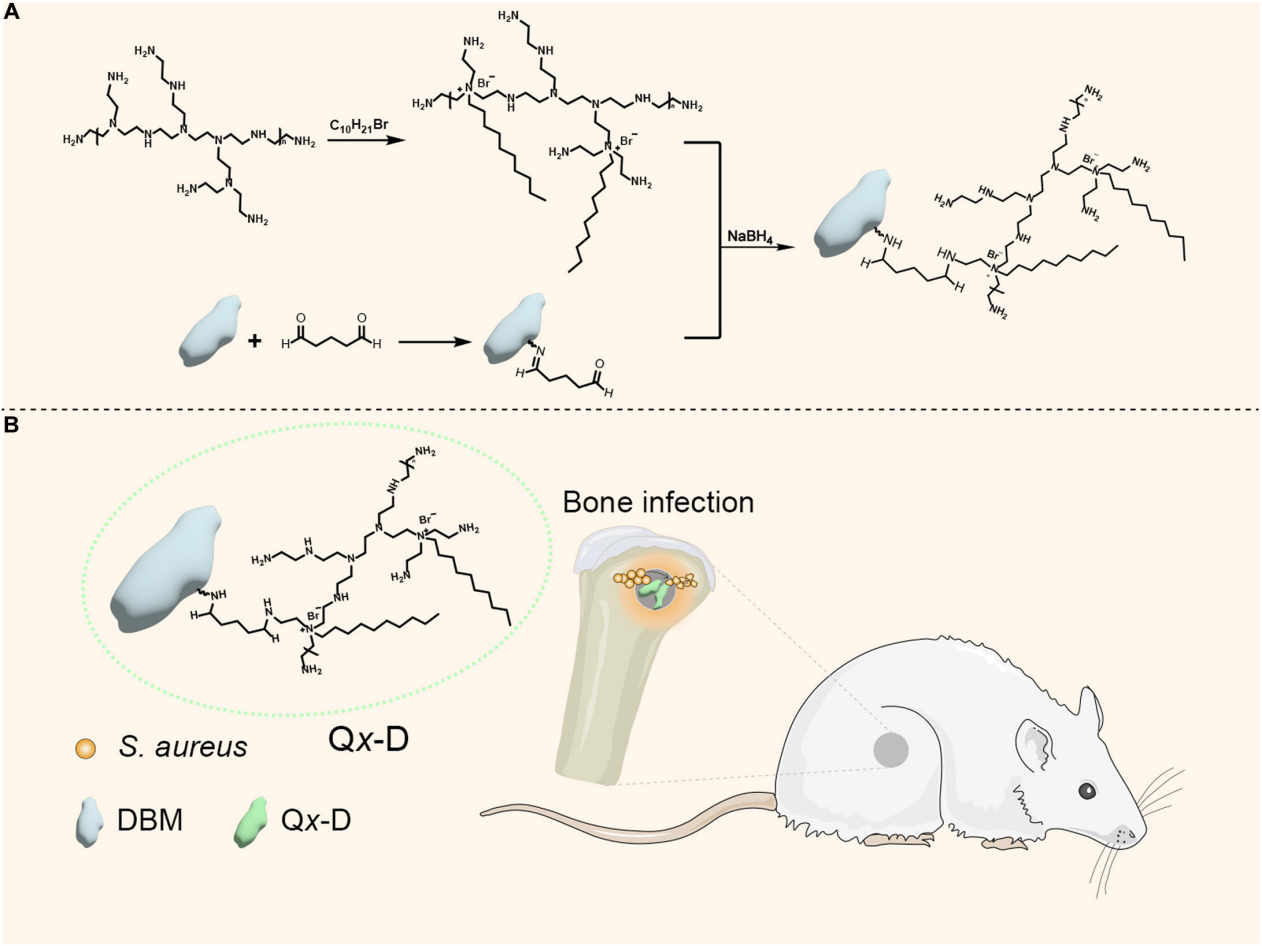
The schematic illustration of the synthesis process of Q*x*-D (A) and the rat model of femoral infectious bone defect (B).

At the present work, an antibacterial functionalization strategy was proposed to avoid the application of antibiotics. DBM was modified with QPEI as an antibacterial component, named Q*x*-D (where *x* = 5, 10, and 15, corresponding to different feeding ratios of QPEI to DBM). Q*x*-D was prepared by using glutaraldehyde as crosslinking component through Schiff base reaction between DBM and QPEI. In order to explore the antimicrobial properties and biocompatibility of Q*x*-D antibacterial tests, hemolysis assay and cytotoxicity assay were performed in vitro. In addition, the anti-infection and osteogenic ability of Q10-D were verified in the rat model of femoral IBD. Finally, the anti-infection and osteointegration-promoting performances of Q10-D in vivo were evaluated. The modified DBM was used to treat drug-resistant bacterial infections without the application of antibiotics. This approach provides a promising method for the development of multifunctional bone regeneration scaffold to address the clinical problems related to IBDs (Fig. 1).

## Results

### Chemical structure analysis of QPEI

To provide an efficient antibacterial agent, QPEI was first synthesized. In order to impart a better antibacterial property, the quaternary ammonium group with alkyl chain containing 10 carbon atoms was introduced into the chemical structure of QPEI [30–33]. The chemical structure and thermal stability of QPEI were analyzed using proton nuclear magnetic resonance spectroscopy (^1^H NMR) and thermogravimetric curve (TG). In the ^1^H NMR spectrum of QPEI (Fig. S1), characteristic peaks were as follows: *δ* = 2.25 to 3.60 parts per million (ppm) (a, N-CH_2_), 1.26 ppm (b, -CH_2_-), and 0.88 ppm (c, -CH_3_). The thermal stability of QPEI was assessed via TG, revealing that QPEI began to decompose at 179 °C and completely decomposed at 397 °C (Fig. S2). These results demonstrated that QPEI was synthesized successfully.

### Physical and chemical properties of DBM and Q*x*-D

**Fig. 2.**
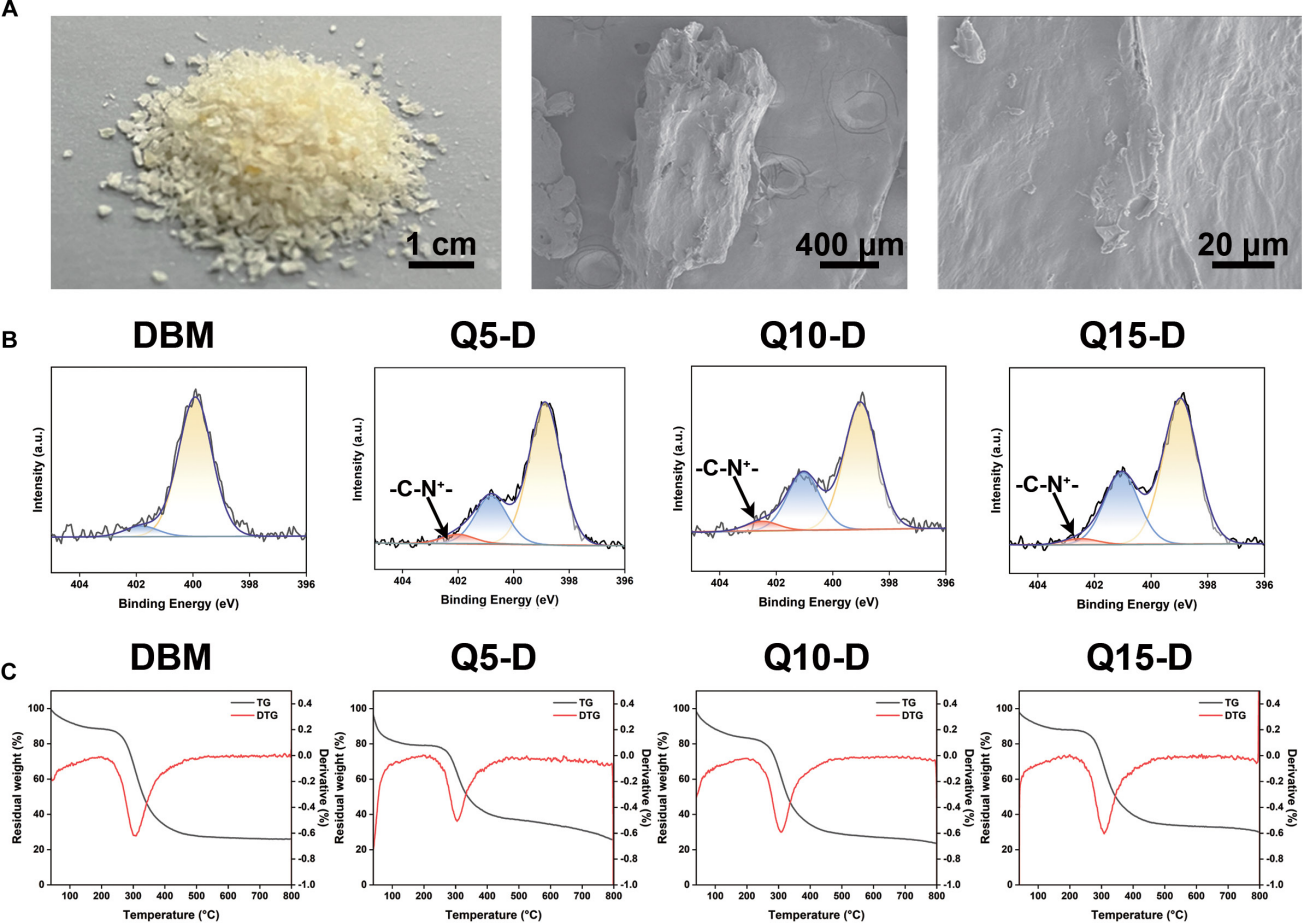
(A) Digital photo and SEM pictures of DBM. (B) High-resolution N 1s spectra of DBM and Q*x*-D were used to assess the successful modification of QPEI and (C) TG of DBM and Q*x*-D could be used to test the thermal stability.

In this study, DBM was obtained from Beijing Tongren Hospital, Capital Medical University. As shown in Fig. 2A, DBM particles were mild yellow. The length and width of DBM particles were approximately 1,500 and 750 μm, respectively, with a uniform size distribution. X-ray photoelectron spectroscopy (XPS), a surface-sensitive analytical technique used to characterize the elemental composition and chemical state of a material, was used to analyze changes in the chemical structure of DBM and Q*x*-D. The XPS spectra of DBM exhibited peaks at O 1s (532 eV), N 1s (399 eV), and C 1s (284 eV). With increasing of QPEI feeding volume (Fig. S3), the intensity of N 1s peak gradually increased, accompanied by a new peak emerging at 402.5 eV. Simultaneously, the shift of the N 1s peak to a higher binding energy (402 eV) confirmed successful QPEI loading (Fig. 2B). Table S1 showed that the atom ratio of N 1s increased from 8.13% to 12. 93% due to the high N atom content of QPEI. XPS and N 1s spectra of DBM and Q*x*-D confirmed the successful modification of QPEI on the surface of DBM. Additionally, TG was used to determine gradient concentrations of QPEI modification on DBM (Fig. 2C). Compared to DBM and QPEI, there was a slight increase in the thermal decomposition temperature of Q*x*-D.

### In vitro antibacterial properties

**Fig. 3.**
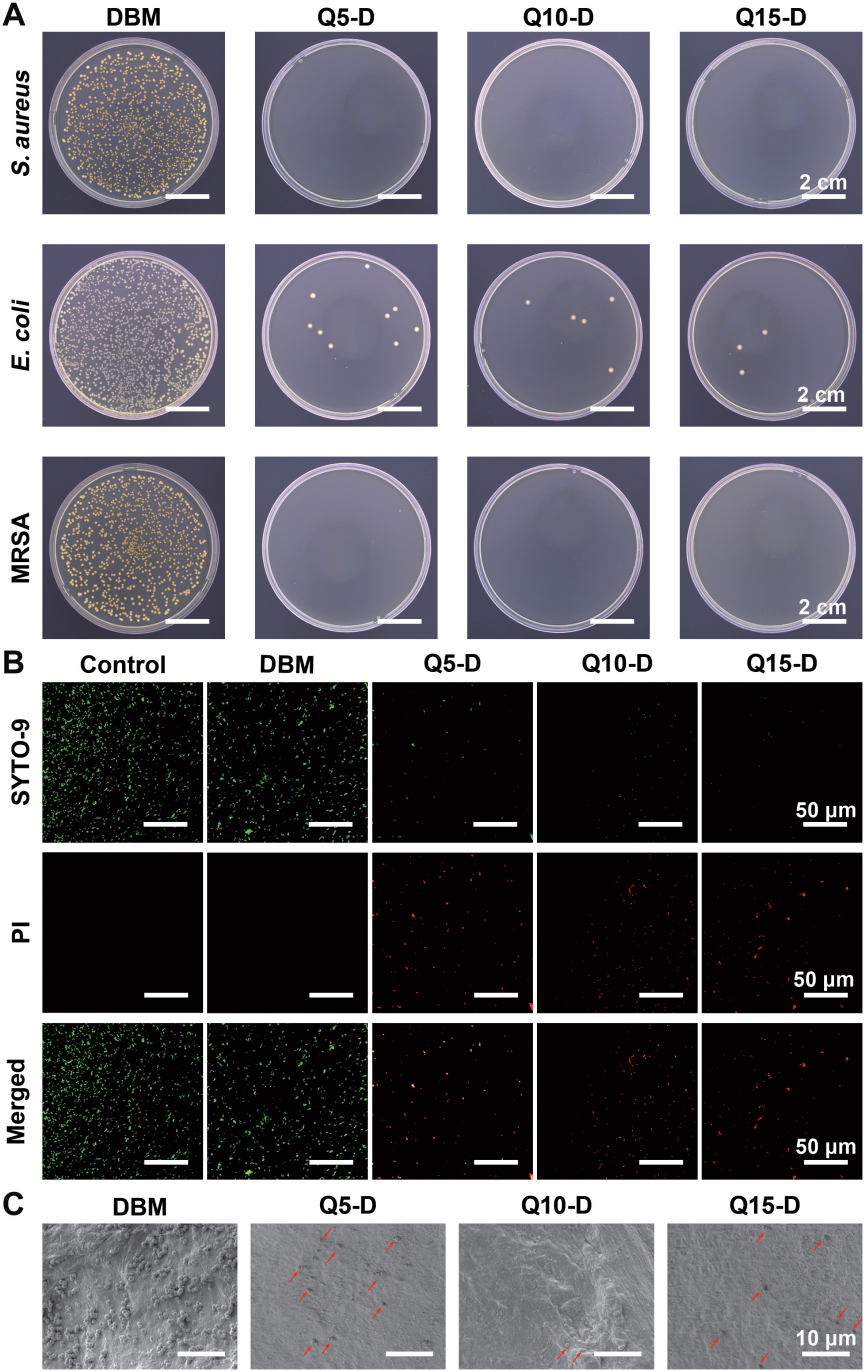
(A) Optical photos of colony count of DBM and Q*x*-D against *S. aureus*, *E. coli*, and MRSA (sample size *n* = 4). (B) CLSM images of DBM and Q*x*-D against *S. aureus* in a simulative severe infection (live and dead bacteria showed green and red fluorescence under laser irradiation, respectively). (C) SEM images of the bacterial adhesion of DBM and Q*x*-D against *S. aureus* (bacteria attached onto surfaces were marked by red arrows).

*S. aureus*, one of the main pathogens that could induct the orthopedic implant-associated infections, was used to test the antibacterial properties of DBM and Q*x*-D. DBM and Q*x*-D were cocultured with *S. aureus* at a density of 10^5^ colony-forming units (CFU)/ml for 12 h. Then, the bacterial suspension was diluted for bacterial culture, and the colonies were counted (Fig. 3A). Compared to DBM, colony counts of all Q*x*-D samples had incredibly decreased. According to the quantitative data in Fig. S4, the antibacterial efficiency of Q*x*-D could reach 99.9%. This demonstrated that Q*x*-D had a significant antibacterial effect against *S. aureus*. When the suspended bacteria came into contact with the Q*x*-D surface, the positive charge carried by QPEI could generate electrostatic attraction with the bacterial cell membrane, causing the alkyl chain to insert into the bacterial cell membrane, thus disturbing and destroying it, ultimately killing the bacteria [31]. This property gave the quaternary ammonium salt, QPEI, certain broad-spectrum antibacterial properties. To further verify whether Q*x*-D possessed broad-spectrum bactericidal properties and could act against drug-resistant bacteria, coculture experiments with *E. coli* and methicillin-resistant *S. aureus* (MRSA) were conducted. The antibacterial efficiency of all Q*x*-D groups against *E. coli* and MRSA also reached 99.9% (Fig. 3A and Fig. S4), indicating that Q*x*-D had broad-spectrum antibacterial activity and was effective against drug-resistant bacteria.

A bacterial suspension of 10^7^ CFU/ml *S. aureus* was cocultured with Q*x*-D to simulate severe infection in vitro. Subsequently, live/dead staining was performed on the bacterial suspension, and confocal laser scanning microscope (CLSM) was used to observe bacterial activity. After staining, live bacteria appeared green and dead bacteria appeared red. The results in Fig. 3B were consistent with the statistical data in Fig. S4. The number of live bacteria in the DBM group was similar to that in the control group, showing no significant antibacterial effect. CLSM images of Q*x*-D revealed a large number of dead bacteria, demonstrating that Q*x*-D modified with QPEI could effectively kill suspended bacteria upon contact with its surface, suggesting the potential efficacy against severe infection.

According to the “race for the surface” theory, the fate of biomaterial implants had been described as a race between microbial adhesion and biofilm growth on the surface of the implants and tissue integration [34]. If the initial infection on the implant surface was not controlled, it would be exacerbated by biofilm formation, affecting the functions of bone-related cells due to bacterial virulence factors and toxins, potentially delaying bone integration [35]. In this process, bacterial adhesion on materials was an important stage [2]. At the same time, bacteria hiding in the pores of the DBM could evade the immune system and contribute to the formation of biofilms [36]. Bacterial adhesion and hiding could become the sources of recurrent infections. To access the ability of Q*x*-D against bacterial adhesion, the surfaces of the samples were observed by scanning electron microscopy (SEM) after coculture antibacterial experiment (Fig. 3C). The results showed that a large number of bacteria adhered to the surface of pristine DBM, while the number of bacteria adhered to the surface of the Q*x*-D group decreased significantly, and the Q10-D group showed the least bacterial adhesion, which was due to the contact killing property of the Q*x*-D samples. These results indicated that Q*x*-D exhibited good antibacterial adhesion properties while effectively killing bacteria, potentially controlling initial infection and enhancing early osseointegration.

### In vitro biocompatibility

**Fig. 4.**
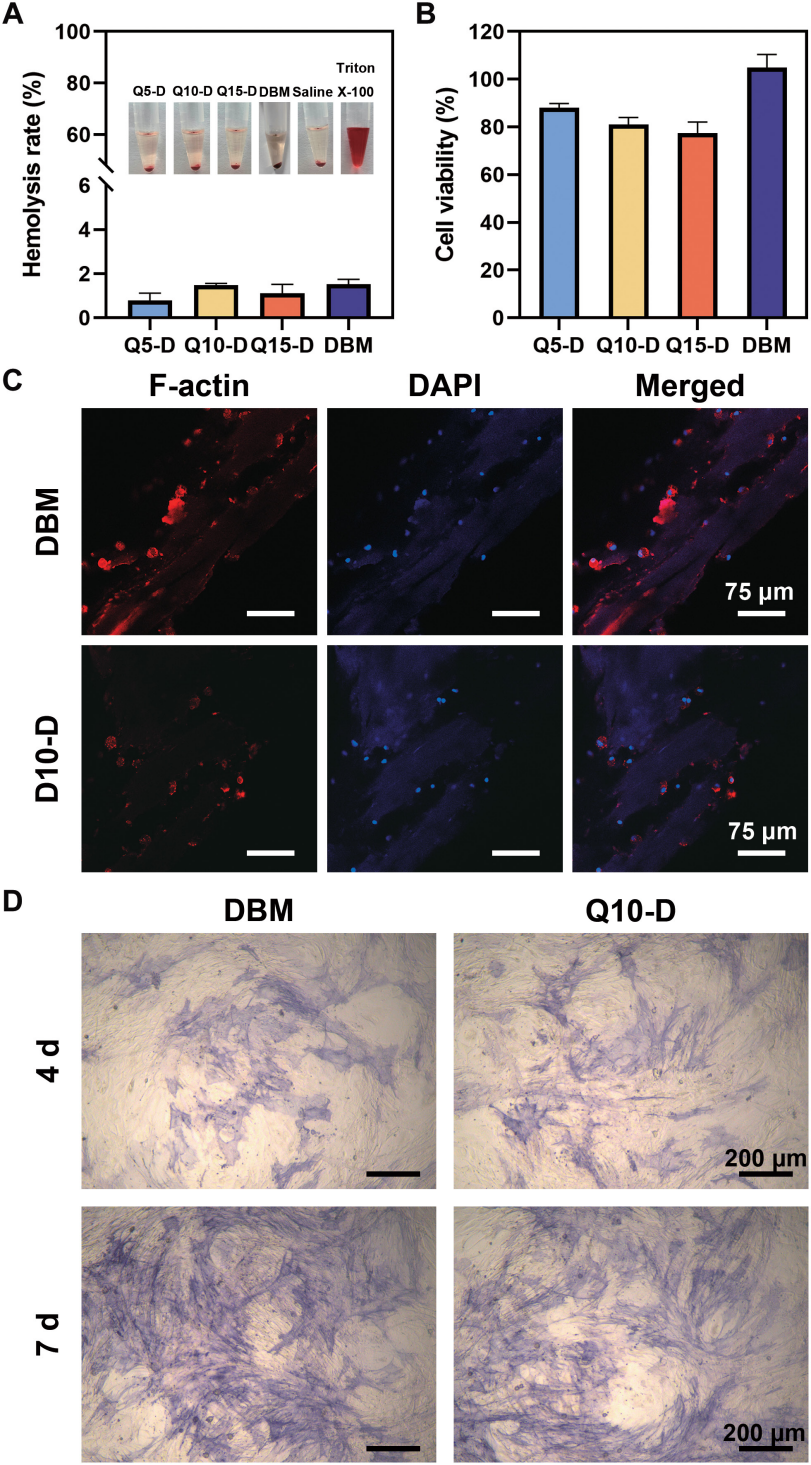
(A) Hemolysis ratio of DBM and Q*x*-D. Triton X-100 and saline were used to set up positive and negative controls, respectively, where 5% was the standard of hemolysis ratio. (B) Cell viability of DBM and Q*x*-D, where 70% was the standard for cell viability. (C) Adhesion of BMSCs on DBM and Q10-D. Rhodamine phalloidin and 4′,6-diamidino-2-phenylindole were used to stain the cytoskeleton and nucleus, respectively, to examine the adhesion of BMSCs on the surface. (D) ALP staining of BMSCs after coculture with DBM and Q10-D. The substrate containing phosphate or phosphate ester can be catalyzed by ALP to produce inorganic phosphate and corresponding color products in alkaline environment.

Biocompatibility was one of the most important characteristics for bone regeneration scaffolds. Hemolysis and cell viability tests were performed to ensure the biocompatibility of Q*x*-D. As shown in Fig. 4A and B, the hemolysis rate of Q*x*-D was less than 5%, and data of cell viability of the Q*x*-D groups were higher than 70%, indicating that DBM modified with QPEI still maintained good biocompatibility. Specifically, the cell activity of the Q*x*-D group decreased with the increasing of QPEI concentration, and the relative cell activity of the Q15 group was slightly lower than that of the Q10 group. This might be due to the Q15 group having the highest feeding ratio of QPEI, resulting in unreacted QPEI being released into the extract. Combined with the results of previous in vitro antibacterial experiments, it was confirmed that Q10-D exhibited excellent antibacterial properties and good biocompatibility. Therefore, Q10-D was selected as the scaffold material for in vivo implantation.

The successful colonization of cells on the surface of implants was conducive to promoting bone integration and osteogenesis. Besides meeting the requirements of biocompatibility, the surface of bone filling materials should also support cell adhesion and proliferation [37]. Therefore, bone marrow mesenchymal stem cells (BMSCs) were inoculated on the surface of DBM and Q10-D and incubated for 24 h. After fluorescence staining, CLSM was used to observe cell adhesion, with results shown in Fig. 4C (red fluorescence representing cytoskeleton and blue fluorescence representing nuclei). CLSM images showed that DBM promoted the adhesion and diffusion of BMSCs, with a large number of cells present on its surface. More BMSCs adhered to the surface of the Q10-D group, indicating that Q10-D could sustain the cell activity of BMSCs. Combined with hemolysis and cytotoxicity tests, the introduction of quaternary ammonium salt did not significantly affect the biocompatibility of DBM, making it a promising candidate for antibacterial bone regeneration scaffold.

Alkaline phosphatase (ALP) was one of the markers of early osteogenic differentiation. To investigate the effects of DBM and Q10-D on osteogenic differentiation of BMSCs, a chromogenic kit was used to detect the expression of ALP in BMSCs after coculture with Q10-D extract [38,39], and the ALP staining results after 4 and 7 d of induction are presented in Fig. 4D. The DBM and Q10-D groups exhibited similar color and similar number of blue-purple nodules, indicating that both groups had a positive bone-promoting effect in vitro, with QPEI showing no significant negative impact on ALP expression in cells. Considering in vitro antibacterial and biocompatibility data, Q10-D demonstrated a favorable balance between antibacterial performance and biocompatibility, establishing a solid foundation for the treatment of IBDs in vivo.

### In vivo anti-infection efficiency

**Fig. 5.**
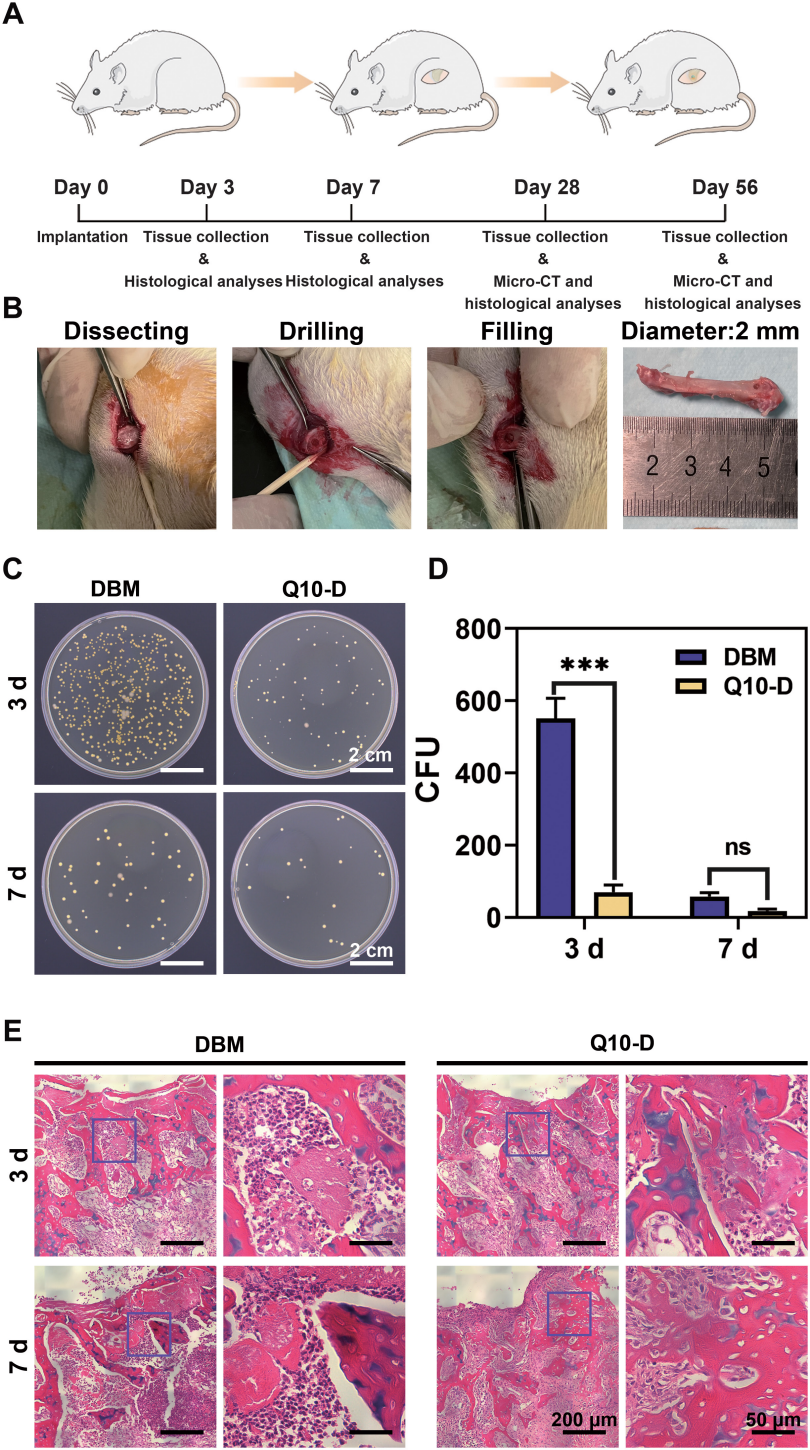
(A) Schematic diagram of an IBD model of rat femur. (B) Digital pictures of the surgery process. (C) Optical photographs and (D) statistics of colony counting (sample size *n* = 3). (E) H&E staining of the bone tissue around the bone defect with Q10-D at 3 and 7 d. H&E can stain the cell nuclei deep blue and the cytoplasm pink, respectively.

The in vitro experimental results proved that Q10-D possessed good antibacterial properties and biocompatibility, and could promote osteogenic differentiation of BMSCs. All of the animal experiments were performed in compliance with the guidelines issued by the Ethical Committee of the Chinese Academy of Sciences. To better address the clinical treatment needs of IBDs, the in vivo anti-infection performances of Q10-D were verified using a rat model with IBDs of femur, as depicted in Fig. 5A. The Q10-D samples infected with *S. aureus* were implanted into the rat femoral defects, with pristine DBM used as the control group. Rat femurs were collected at 3 and 7 d after surgery (Fig. 5B). The implanted samples were removed from the femurs and soaked in phosphate-buffered saline for bacterial culture, and the numbers of bacterial colonies were counted in Fig. 5C. According to the statistical results in Fig. 5D, the number of colonies in the Q10-D group at 3 d was significantly lower than that in the DBM group. At 7 d, both the DBM and Q10-D groups showed significantly fewer colonies compared to the results at 3 d. Additionally, the number of colonies in the Q10-D group remained lower than that in the DBM group, indicating that Q10-D could effectively kill bacteria attached to the surface in the early stage. Tissue inflammation in rats was evaluated through tissue section staining. Hematoxylin and eosin (H&E) staining of the bone tissues surrounding the defect was performed at 3 and 7 d after operation. As shown in Fig. 5E, a large number of inflammatory cells (neutrophils, lymphocytes, etc.) were observed in the DBM group at both 3 and 7 d. However, the number of inflammatory cells in the Q10-D group was significantly lower than that in the DBM group, indicating that Q10-D implantation could effectively reduce bone defect infection and inflammation.

### In vivo osteogenic activity

**Fig. 6.**
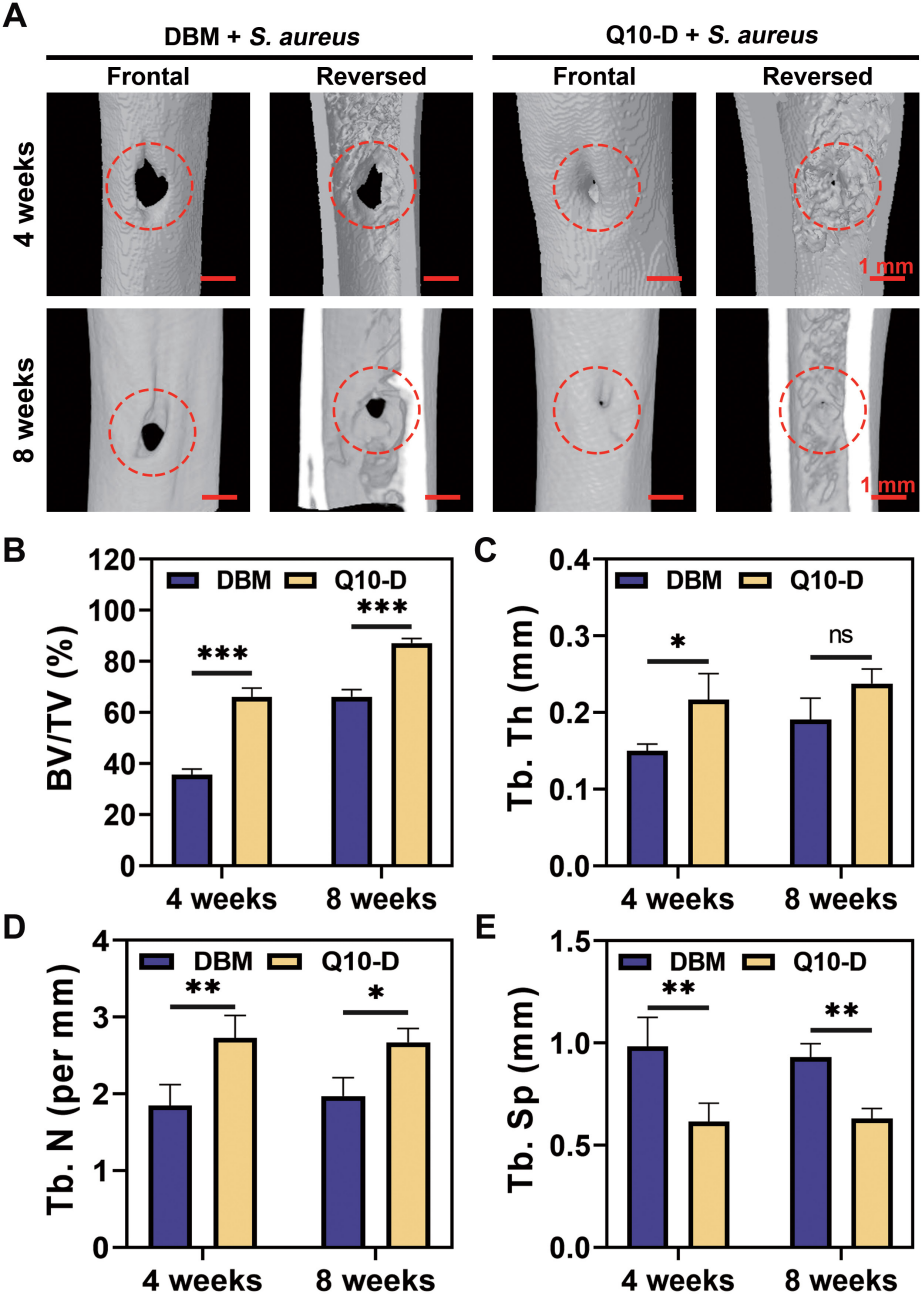
Micro-CT images of a *S. aureus*-infected bone defect model of rat femur for 4 and 8 weeks (A) and quantitative statistics (B to E) of DBM and Q10-D. The red circles marked the location of the bone defect. Sample size *n* = 3 for each parameter.

Q10-D was implanted into the femoral defect of rats to explore its in vivo osteogenic ability. Three-dimensional reconstructed micro-computed tomography (CT) images were used to analyze the collected bone tissue. The results are shown in Fig. 6A, where the original bone defect (diameter = 2 mm) was marked in the red circle. As seen in the micro-CT images, bone defects were still observed in the DBM + *S. aureus* group at 4 weeks after surgery, whereas the defect area in the Q10-D + *S. aureus* group was significantly reduced, indicating that the bone tissue recovered obviously. This might be attributed to bacteria adhering to and proliferating in large numbers on the surface of DBM, releasing virulence factors and toxins that could affect the surrounding cells, leading to bone tissue necrosis (*32*). Q10-D exhibited antibacterial properties and anti-bacterial adhesion properties, which can reduce the negative impact of bacterial infection on bone healing. At 8 weeks after surgery, new bone was also produced in the DBM + *S. aureus* group, whereas the defect was nearly completely closed in the Q10-D + *S. aureus* group.

Furthermore, quantitative analysis of new bone tissue was presented in Fig. 6B to E. The ratio of bone tissue volume to total tissue volume, denoted as BV/TV, is defined as the proportion of bone tissue volume relative to the total tissue volume within a specified area. This ratio serves as a common metric for evaluating bone density and overall bone health. In the Q10-D group, the BV/TV ratio reached approximately 66% 4 weeks after surgery, which was notably higher compared to the DBM group, where the BV/TV ratio was around 35%. At 8 weeks after surgery, BV/TV in the Q10-D group reached about 87%, still significantly higher than that in the DBM group. In addition, trabecular number (Tb. N) in the Q10-D + *S. aureus* group was significantly higher than that in the DBM + *S. aureus* group at 4 and 8 weeks after surgery, while trabecular separation (Tb. Sp) was significantly lower than that in the DBM + *S. aureus* group. These results suggested that Q10-D significantly enhanced bone regeneration in the presence of bacterial infection compared to DBM.

**Fig. 7.**
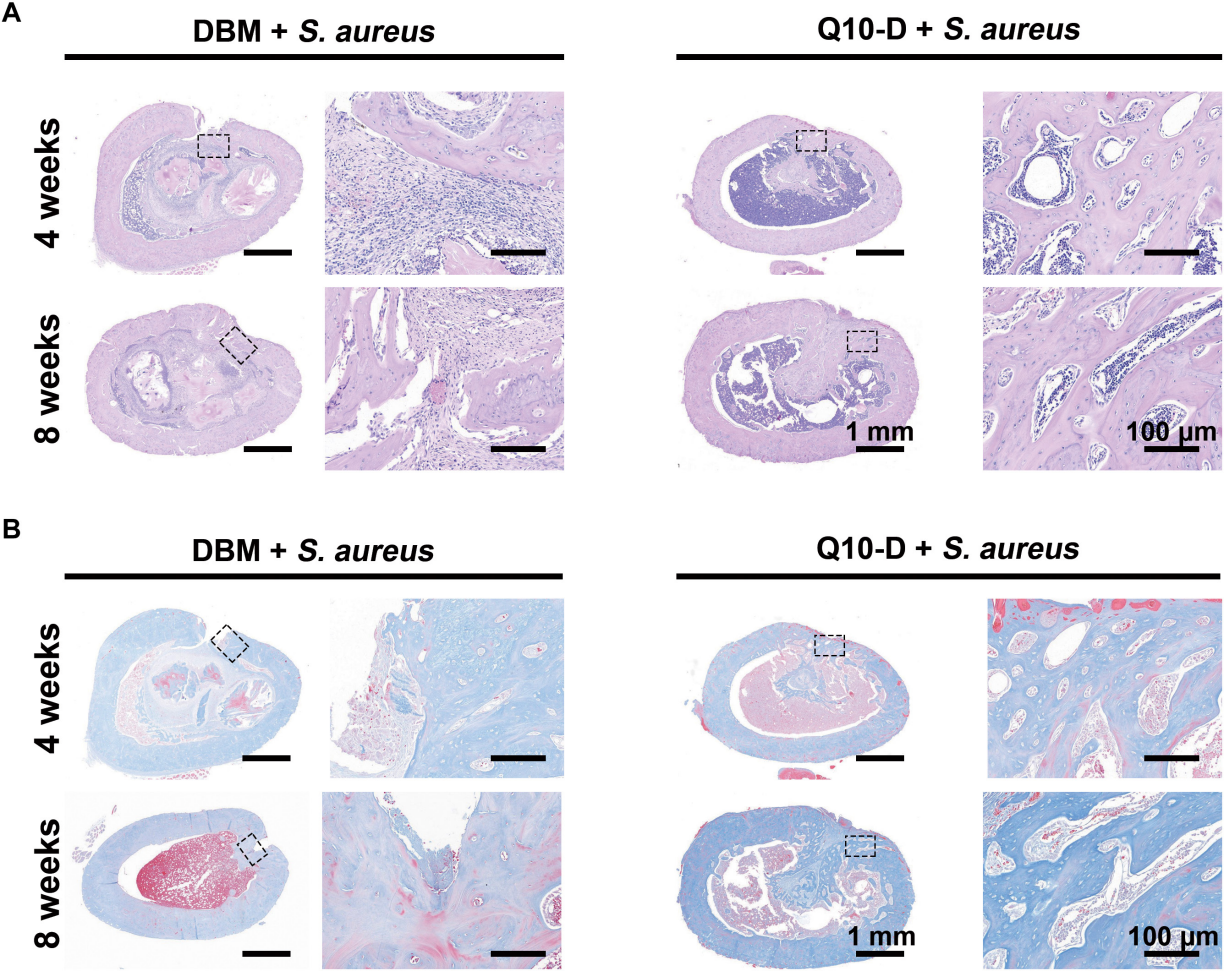
(A) H&E staining and (B) Masson’s staining of bone tissue at 4 and 8 weeks after surgery.

Infection and osteogenesis were further evaluated using H&E and Masson’s staining (Fig. 7), respectively. At 4 weeks after surgery, the DBM + *S. aureus* group exhibited obvious inflammatory cell infiltration, whereas the number of inflammatory cells was significantly reduced in the Q10-D + *S. aureus* group. The inflammatory cell infiltration in the DBM + *S. aureus* group at 8 weeks after surgery slightly decreased compared to that at 4 weeks, while the number of inflammatory cells in the Q10-D + *S. aureus* group remained much smaller than that in the DBM + *S. aureus* group. Masson’s staining revealed that new bone formation in the Q10-D + *S. aureus* group was significantly better than that in the DBM + *S. aureus* group at 4 and 8 weeks after surgery. In addition, most of the new bone formed in the Q10-D + *S. aureus* group was mature and fully mineralized bone (blue stain), whereas very little mature bone formation was observed in the DBM + *S. aureus* group. The above results showed that Q10-D could effectively clear *S. aureus* in bone defects and reduce the inflammation reaction.

## Discussion

In summary, we proposed an efficient approach for antibacterial functionalization of DBM. Q*x*-D had broad-spectrum antibacterial performance (>99.99%) against *S. aureus*, *E. coli*, and MRSA. In addition, all Q*x*-D samples exhibited extremely low hemolysis ratio (<5%) and high cell viability (>70%) in vitro. The results of in vivo experiments showed that Q10-D showed good antibacterial properties at early stage after surgery. Micro-CT images and statistical data demonstrated the remarkable osteogenic ability of Q10-D at 4 weeks after surgery. Finally, H&E staining and Masson’s staining were applied to prove the good bone-repairing performances of Q10-D. This work provided a promising strategy for constructing functional surfaces with balanced bactericidal and osteogenic properties for development of novel orthopedic implants.

## Materials and Methods

The materials and methods were described in the Supplementary Materials.

## Data Availability

All data are available from the authors upon request.

## References

[1] Habibovic P. Strategic directions in osteoinduction and biomimetics. Tissue Eng A. 2017;23(23–24):1295–1296. 10.1089/ten.TEA.2017.0430 29032745

[2] Arciola CR, Campoccia D, Montanaro L. Implant infections: Adhesion, biofilm formation and immune evasion. Nat Rev Microbiol. 2018;16:397–409. 29720707 10.1038/s41579-018-0019-y

[3] Sarkissian EJ, Gans I, Gunderson MA, Myers SH, Spiegel DA, Flynn JM. Community-acquired methicillin-resistant Staphylococcus aureus musculoskeletal infections: Emerging trends over the past decade. J Pediatr Orthop. 2016;36(3):323–327. 25785593 10.1097/BPO.0000000000000439

[4] Lebowitz D, Kressmann B, Gjoni S, Zenelaj B, Grosgurin O, Marti C, Zingg M, Uçkay I. Clinical features of anaerobic orthopaedic infections. Infect Dis. 2017;49(2):137–140. 10.1080/23744235.2016.1225979 27581503

[5] Ghosh S, Sinha M, Samanta R, Sadhasivam S, Bhattacharyya A, Nandy A, Saini S, Tandon N, Singh H, Gupta S, et al. A potent antibiotic-loaded bone-cement implant against staphylococcal bone infections. Nat Biomed Eng. 2022;6:1180–1195. 36229662 10.1038/s41551-022-00950-x PMC10101771

[6] Lin Z, Zhao Y, Chu PK, Wang L, Pan H, Zheng Y, Wu S, Liu X, Cheung KMC, Wong T, et al. A functionalized TiO_2_/Mg_2_TiO_4_ nano-layer on biodegradable magnesium implant enables superior bone-implant integration and bacterial disinfection. Biomaterials. 2019;219: Article 119372. 31362176 10.1016/j.biomaterials.2019.119372

[7] Afewerki S, Bassous N, Harb S, Palo-Nieto C, Ruiz-Esparza GU, Marciano FR, Webster TJ, Furtado ASA, Lobo AO. Advances in dual functional antimicrobial and osteoinductive biomaterials for orthopaedic applications. Nanomedicine. 2020;24: Article 102143. 31862427 10.1016/j.nano.2019.102143

[8] Ansari M. Bone tissue regeneration: Biology, strategies and interface studies. Prog Biomater. 2019;8(4):223–237. 31768895 10.1007/s40204-019-00125-z PMC6930319

[9] Morris AH, Stamer DK, Kyriakides TR. The host response to naturally-derived extracellular matrix biomaterials. Semin Immunol. 2017;29:72–91. 28274693 10.1016/j.smim.2017.01.002

[10] Alom N, Peto H, Kirkham GR, Shakesheff KM, White LJ. Bone extracellular matrix hydrogel enhances osteogenic differentiation of C2C12 myoblasts and mouse primary calvarial cells. J Biomed Mater Res B Appl Biomater. 2018;106(2):900–908. 28429412 10.1002/jbm.b.33894

[11] Gothard D, Smith EL, Kanczler JM, Black CR, Wells JA, Roberts CA, White LJ, Qutachi O, Peto H, Rashidi H, et al. In vivo assessment of bone regeneration in alginate/bone ECM hydrogels with incorporated skeletal stem cells and single growth factors. PLOS ONE. 2015;10(12): Article e0145080. 26675008 10.1371/journal.pone.0145080 PMC4684226

[12] Mansour A, Mezour MA, Badran Z, Tamimi F. Extracellular matrices for bone regeneration: A literature review. Tissue Eng A. 2017;23(23–24):1436–1451. 10.1089/ten.TEA.2017.0026 28562183

[13] Han L, Yuan Z, Ren HM, Song W, Wu R, Li J, Guo Z, Yu B, Duan S, Xu FJ. Infection-responsive polysaccharide-based drug-loaded nano-assembly for dual-modal treatment against drug-resistant bacterial lung infection. BMEMat. 2024; Article e12082.

[14] Shariatinia Z, Karimzadeh Z. Perovskite oxides as efficient bioactive inorganic materials in tissue engineering: A review. Mater Today Chem. 2024;35: Article 101846.

[15] Madiwal V, Khairnar B, Rajwade J. Enhanced antibacterial activity and superior biocompatibility of cobalt-deposited titanium discs for possible use in implant dentistry. iScience. 2024;27(2):108827. 38303692 10.1016/j.isci.2024.108827 PMC10831949

[16] Chen X, Sun M, Zhang L, Hu Y, Yang Z, Duan S, Xu FJ, Jing J. A one-step polyphenol-based functionalization strategy of dual-enhanced antibacterial and osteogenic surfaces. Chem Eng J. 2024;490: Article 151792.

[17] Tsave O, Iordanidou C, Hatzidimitriou A, Yavropoulou MP, Kassi EN, Nasiri-Ansari N, Gabriel C, Salifoglou A. Structural speciation of Ti(IV)-(α-hydroxycarboxylic acid) complexes in metabolism-related (patho)physiology—In vitro approaches to (pre)adipocyte differentiation and mineralization. Int J Mol Sci. 2023;24(14):11865. 37511624 10.3390/ijms241411865 PMC10380816

[18] Zhang Z, Liu A, Fan J, Wang M, Dai J, Jin X, Deng H, Wang X, Liang Y, Li H, et al. A drug-loaded composite coating to improve osteogenic and antibacterial properties of Zn–1Mg porous scaffolds as biodegradable bone implants. Bioact Mater. 2023;27:488–504. 37180641 10.1016/j.bioactmat.2023.04.017 PMC10173180

[19] Jia C, Wu F-G. Antibacterial chemodynamic therapy: Materials and strategies. BME Front. 2023;4:0021. 37849674 10.34133/bmef.0021 PMC10351393

[20] Qian Y, Qi F, Chen Q, Zhang Q, Qiao Z, Zhang S, Wei T, Yu Q, Yu S, Mao Z, et al. Surface modified with a host defense peptide-mimicking β-peptide polymer kills bacteria on contact with high efficacy. ACS Appl Mater Interfaces. 2018;10(18):15395–15400. 29688003 10.1021/acsami.8b01117

[21] Adekoya GJ, Ezika AC, Adekoya OC, Sadiku ER, Hamam Y, Ray SS. Recent advancements in biomedical application of polylactic acid/graphene nanocomposites: An overview. BMEMat. 2023;1(4): Article e12042.

[22] Chen L, Ren HM, Sun Y, Li J, Guo Z, Yu B, Ding X, Duan S, Xu FJ. A dual-enhancement antibacterial strategy for hierarchically functionalized surfaces with photodynamic and antifouling performance. Mater Today Chem. 2024;36: Article 101945.

[23] Ma L, Zong J, Xun X, Hu X, Chen Z, Zhang Q, Peng M, Song B, Ao H. Fabrication of gentamicin loaded Col-I/HA multilayers modified titanium coatings for prevention of implant infection. Front Chem. 2022;10:1019332. 36482941 10.3389/fchem.2022.1019332 PMC9722959

[24] Cao H, Qiao S, Qin H, Jandt KD. Antibacterial designs for implantable medical devices: Evolutions and challenges. J Funct Biomater. 2022;13(3):86. 35893454 10.3390/jfb13030086 PMC9326756

[25] Liu X, Jiang Z, Xing D, Yang Y, Li Z, Sun Z. Recent progress in nanocomposites of carbon dioxide fixation derived reproducible biomedical polymers. Front Chem. 2022;10:1035825. 36277338 10.3389/fchem.2022.1035825 PMC9585172

[26] Fang B, Qiu P, Xia C, Cai D, Zhao C, Chen Y, Wang H, Liu S, Cheng H, Tang Z, et al. Extracellular matrix scaffold crosslinked with vancomycin for multifunctional antibacterial bone infection therapy. Biomaterials. 2021;268: Article 120603. 33378735 10.1016/j.biomaterials.2020.120603

[27] Sun Y, Zhao YQ, Zeng Q, Wu YW, Hu Y, Duan S, Tang Z, Xu FJ. Dual-functional implants with antibacterial and osteointegration-promoting performances. ACS Appl Mater Interfaces. 2019;11(40):36449–36457. 31532178 10.1021/acsami.9b14572

[28] Zhou Y, Deng J, Zhang Y, Li C, Wei Z, Shen J, Li J, Wang F, Han B, Chen D, et al. Engineering DNA-guided hydroxyapatite bulk materials with high stiffness and outstanding antimicrobial ability for dental inlay applications. Adv Mater. 2022;34(27):2202180. 10.1002/adma.202202180 35488765

[29] Teotia A, Laurén I, Borandeh S, Seppälä J. Quaternized chitosan derivatives as viable antiviral agents: Structure–activity correlations and mechanisms of action. ACS Appl Mater Interfaces. 2023;15(15):18707–18719. 37014147 10.1021/acsami.3c01421 PMC10119858

[30] Zheng Z, Xu Q, Guo J, Qin J, Mao H, Wang B, Yan F. Structure–antibacterial activity relationships of imidazolium-type ionic liquid monomers, poly(ionic liquids) and poly(ionic liquid) membranes: Effect of alkyl chain length and cations. ACS Appl Mater Interfaces. 2016;8(20):12684–12692. 27145107 10.1021/acsami.6b03391

[31] Jiao Y, Niu LN, Ma S, Li J, Tay FR, Chen JH. Quaternary ammonium-based biomedical materials: State-of-the-art, toxicological aspects and antimicrobial resistance. Prog Polym Sci. 2017;71:53–90. 32287485 10.1016/j.progpolymsci.2017.03.001 PMC7111226

[32] Jain A, Duvvuri LS, Farah S, Beyth N, Domb AJ, Khan W. Antimicrobial polymers. Adv Healthc Mater. 2014;3(12):1969–1985. 25408272 10.1002/adhm.201400418

[33] Lin J, Qiu S, Lewis K, Klibanov AM. Mechanism of bactericidal and fungicidal activities of textiles covalently modified with alkylated polyethylenimine. Biotechnol Bioeng. 2003;83(2):168–172. 12768622 10.1002/bit.10651

[34] Gristina AG. Biomaterial-centered infection: Microbial adhesion versus tissue integration. Science. 1987;237(4822):1588–1595. 3629258 10.1126/science.3629258

[35] Li Z, Zhang S, Fu Z, Liu Y, Man Z, Shi C, Tang C, Chen C, Chai Q, Yang Z, et al. Surficial nano-deposition locoregionally yielding bactericidal super CAR-macrophages expedites periprosthetic osseointegration. Sci Adv. 2023;9:eadg3365. 37256944 10.1126/sciadv.adg3365 PMC10413653

[36] de Mesy Bentley KL, Trombetta R, Nishitani K, Bello-Irizarry SN, Ninomiya M, Zhang L, Chung HL, McGrath JL, Daiss JL, Awad HA, et al. Evidence of staphylococcus aureus deformation, proliferation, and migration in canaliculi of live cortical bone in murine models of osteomyelitis. J Bone Miner Res. 2017;32(22):985–990. 27933662 10.1002/jbmr.3055 PMC5413415

[37] Li J, Ma J, Sun H, Yu M, Wang H, Meng Q, Li Z, Liu D, Bai J, Liu G, et al. Transformation of arginine into zero-dimensional nanomaterial endows the material with antibacterial and osteoinductive activity. Sci Adv. 2023;9(21):eadf8645. 37235658 10.1126/sciadv.adf8645 PMC10219602

[38] Lu G, Zhao G, Wang S, Li H, Yu Q, Sun Q, Wang B, Wei L, Fu Z, Zhao Z, et al. Injectable nano-micro composites with anti-bacterial and osteogenic capabilities for minimally invasive treatment of osteomyelitis. Adv Sci. 2024;11:2306964. 10.1002/advs.202306964 PMC10966557 38234236

[39] Ge Y, Wang K, Liu J, Tian Y, Li H, Wang HZ, Lin Z, Qiu M, Tang B. A ZIF-8-based multifunctional intelligent drug release system for chronic osteomyelitis. Colloids Surf B Biointerfaces. 2022;212: Article 112354. 35085938 10.1016/j.colsurfb.2022.112354

