# Supplementary material for: Cationized Decalcified Bone Matrix for Infected Bone Defect Treatment
Source: BME Front. 2024 Oct 2;5:0066. doi: 10.34133/bmef.0066 (PMC11445788; doi:10.34133/bmef.0066)
Supplement: Supplementary 1 — Materials and Methods Figs. S1 to S4 Table S1 [file bmef.0066.f1.zip › Supplementary Materials.docx]

**Supplementary Materials**

**Cationized decalcified bone matrix for infected bone defect treatment**

Le Chen,^1,#^ Yuying Ai,^1,#^ Ruonan Wu,^1^ Zhaoyan Guo,^3^ Yang Li,^1^ Jie Li,^3,*^ Feng Qu,^2,*^ Shun Duan,^1,*^ Fu-Jian Xu^1,*^

^1^State Key Laboratory of Chemical Resource Engineering, Key Lab of Biomedical Materials of Natural Macromolecules (Beijing University of Chemical Technology), Ministry of Education, Beijing Laboratory of Biomedical Materials, Beijing University of Chemical Technology, Beijing 100029, China

2Beijing Tongren Hospital, Capital Medical University, Beijing 100730

^3^Beijing Research Institute of Chemical Industry, Sinopec, Beijing 100013

^*^To whom all correspondence should be addressed:

E-mail: lijie.bjhy@sinopec.com (J Li); qforthop@163.com (F Qu); duanshun@mail.buct.edu.cn (S Duan); xufj@mail.buct.edu.cn (F J Xu).

^#^These authors contributed equally to this work.

1. **Experimental section**

**1.1. Materials.** Demineralized bone matrix (DBM), was provided by Beijing Tongren Hospital, Capital Medical University. Polyethyleneimine (PEI, *M*_w_~1800 g/mol, 99%, provided by Shanghai Dibai Biotechnology Co., Ltd), 1-bromodecane (C_10_H_21_Br, 98%, provided by Energy Chemical), *N*,*N*-dimethylformamide (DMF, 99.9%, provided by Shanghai Macklin Biochemical Technology Co., Ltd), sodium carbonate (Na_2_CO_3_, AR, ≥99.8%, provided by Energy Chemical), chloroform-d (CDCl_3_, provided by Energy Chemical), glutaraldehyde (GA, 50% in water, provided by Energy Chemical), sodium borohydride (NaBH_4_, AR, ≥98%, provided by Beijing Tong Guang Fine Chemicals Company), , ethanol (AR, provided by Beijing Chemical Plant) were used without any other treatment..

**1.2. Preparation of the quaternized polyethyleneimine (QPEI).** QPEI was synthesized by quaternization reaction of PEI with C_10_H_21_Br. 5 g PEI was dissolved in 10 mL DMF in a 50 mL beaker. 10 mL C_10_H_21_Br was dissolved in 10 mL DMF in a 100 mL flask. Then PEI solution was slowly dropped into C_10_H_21_Br solution, and the mixed solution was reacted at 50℃ for 24 h. 3g Na_2_CO_3_ was added into the flask in order to stop the reaction. The product was precipitated by n-hexane, transferred to a dialysis bag (1000 Da) for dialysis with deionized water for 3 days, lyophilized and stored at -20℃. The quaternization ratio of PEI was 73% by ^1^H NMR (Fig. S1).

**1.3. Preparation of DBM modified with QPEI (Q*x*-D).** DBM was modified with QPEI through covalent attachment by GA. 50 mg DBM was added in 10 mL GA (2.5% v/v) aqueous solution in a 20 mL sample vial, and the mixture was magnetically stirred at room temperature for 3 hours. Remove the supernatant. The DBM modified GA was immersed in 5 mL QPEI solution (pH=7.4) at gradient concentrations (5, 10, 15 mg/mL, w/v) for 5 h. After the reaction, the materials were immersed in 5 mL NaBH_4_ solution (3 mg/mL, w/v) for 2 h. Finally, prepared materials were washed three times with normal saline under ultrasound and dried in 37℃ vacuum drying oven. The samples obtained from different reaction concentrations were named Q*x*-D (*x*=5,10,15).

**1.4. Characterization.** The surface morphology of DBM was observed by optical microscopy and scanning electron microscopy (SEM, JSM-7500F, JEOL, Japan). The surface chemical compositions of Q*x*-D were characterized by X-ray photoelectron spectroscopy (XPS, Kratos AXIS-His, Shimadzu, Japan). The thermal stability of Q*x*-D was measured by thermal gravimetric analysis (TGA, TG209F3Tarsus, Netzsch, Germany), with a heating rate of 10 °C/min from 40 °C to 800 °C.

**1.5. *In vitro* antibacterial properties.** The *in* *vitro* antibacterial properties were first tested*.* *S. aureus*, *E. coli* and methicillin-resistant *staphylococcus aureus* (MRSA*)* were used to investigate the antibacterial activity of various specimens *in vitro*. *S. aureus*, *E. coli* and MRSA were grown in Luria Broth (LB) medium to a density of 0.6 to 0.8 × 10^8^ CFU/mL. The density of bacterial suspensions was diluted to 10^5^ and 10^7^ CFU/mL in LB for *in vitro* antibacterial study. 5 mg of DBM and Q*x*-D, which were sterilized by ultraviolet, were placed in a 48-well plate. Then, 500 μL of 10^5^ CFU/mL bacterial suspension was added into each well, and incubated at 37 ℃ for 12 h. After incubation, 100 μL of the supernatant of each well was taken to count the bacterial content by spread-plate method (SPM).

To simulate severe infection, 10^7^ CFU/mL of *S. aureus* suspension was co-incubated with DBM and Q*x*-D for 12 h. After the co-incubation, the bacteria in the supernatant were stained by SYTO-9/PI, followed by observation of the survival and death of bacteria by using confocal laser scanning microscopy (CLSM, TCS SP8, Leica, Germany). The bacterial morphology and quantity on surfaces of DBM and Q*x*-D were observed by SEM. Bacteria on the surface of DBM and Q*x*-D were fixed with 2.5% GA at 4 ℃ for 8 h, dehydrated with gradient ethanol (30%, 50%, 70%, 80%, 90%, 95%, 100% and 100%), and observed by SEM.

**1.6. *In vitro* biocompatibility.** *1.6.1 Hemolysis assay.* 10 mL normal saline was added to 1 mL fresh blood from rat, and centrifuged at 2000 rpm for 15 mins. The supernatant was discarded and the whole blood was washed for three times following the same steps. The red blood cell was prepared into 2% suspension with normal saline. Triton X-100 and normal saline were added into 1 mL 2% red blood cell suspension as a positive control group and a negative control group, respectively. 5 mg of DBM and Q*x*-D were put into a 48-well plate, respectively. 500 μL 2% red blood cell solution was added into each well. The plate was incubated at 37℃ for 3 h. The solution was added into 1.5 mL centrifuge tube, and centrifuged at 2000 rpm for 15 min. 100 μL supernatant was added into 96-well plate, and measured the absorbance at 545 nm by the microplate reader.

*1.6.2 Cell Viability Assay.* In addition, cytotoxicity was also an important factor in evaluating the biocompatibility of biomedical materials. Mouse fibroblasts (L929) were selected to evaluate *in vitro* biocompatibility of DBM and Q*x*-D. The cells were cultured in RPMI 1640 medium (Gibco, USA) containing 10% fetal bovine serum (FBS, Gibco, USA) and 1% penicillin-streptomycin (Gibco, USA). When the cell density reached 80%, the cells were diluted to 10^5^ cells/mL using the above culture medium. DBM and Q*x*-D were sterilized by ultraviolet in advance, and then placed into 96-well plate. The cell dilution was added into each well, and incubated in incubator for 24 h. Three parallel samples s were set for each implant.

The cell viability was measured by MTT assay. 5 mg MTT was dissolved into 1 mL PBS buff solution and 9 mL RPMI 1640 medium in dark environment. The original solution was replaced by equal volume of MTT solution. The plate was incubated for another 4 h, and replaced by equal volume of DMSO. 100 μL solution was added into a 96-well plate and measured the absorbance at 570 nm by the microplate reader (Cytation 3, Biotek, USA). The relative cell survival rate is calculated as follows:

$$Cell Viability \left( \% \right)=\frac{{OD}_{test}-{OD}_{neg}}{{OD}_{test}-{OD}_{pos}}\times100\%$$

*1.6.3 Fluorescent staining.* BMSCs were cultured in α-minimum essential medium (α-MEM, Gibco, USA) containing 10% FBS and 1% penicillin-streptomycin. BMSCs were seeded on DBM and Q*x*-D and cultured for 24 h. Then, they were fixed with 4% paraformaldehyde for 20 min at room temperature. Afterward, the cells were washed with PBS and sealed and permeabilized with 3% bovine serum albumin solution containing 0.3% Triton X-100 for 12 h. DBM and Q*x*-D were stained with rhodamine phalloidin and 4′,6-diamidino-2-phenylindole. Images were acquired with an inverted fluorescence microscope.

*1.6.4 ALP staining.* BMSCs were seeded in a 24-well plate at a density of 10^4^ cells per well. After 24 h, the medium was replaced with osteogenic differentiation medium (β-glycerophosphate: 10 mM, ascorbic acid: 300 nM, dexamethasone: 10 nM). UV sterilized DBM and Q10-D were immersed in the osteogenic induction medium mentioned above, with the medium being changed every 2 days. After 4 or 7 days of cultivation, the cells were stained using an ALP staining kit (Beyotime, China), and images were captured under an optical microscope.

**1.7. *In vivo* antibacterial properties.** All of the animal experiments were performed in compliance with the guidelines issued by the Ethical Committee of the Chinese Academy of Sciences. An infected bone defect model was made on male Sprague-Dawley rats (weight ranges 310 to 360 g). The rats were randomly divided into 2 groups, named the DBM group and the Q10-D group, with 6 rats in each group. After UV sterilization, samples of DBM and Q10-D were soaked in a suspension of 10^8^ CFU/mL *S. aureus* for 30 min. Following anesthesia with isoflurane, the hair on the right hind limb of the rats was shaved and disinfected with iodine. The femur was exposed by blunt dissection, and a bone defect was created at the distal end of the femur using a bone drill (diameter: 2 mm, depth: 2 mm). Subsequently, the DBM and Q10-D soaked in the bacterial suspension were implanted into the bone defect of the femur.

To investigate the infection status at the femur of rats, three rats from each group were sacrificed at 3- or 7-days post-operation to harvest femoral tissues and extract the implanted materials from the defects. The retrieved materials were promptly soaked in 1 mL of sterile PBS for 12 h. After soaking, 50 μL of thse leach liquor was spread to a standard agar culture plate for further incubation at 37°C for 24 h，and the bacterial colonies were counted after incubation. The collected femoral specimens were fixed in 4% paraformaldehyde, followed by decalcification in JYBL-1 decalcification solution for 24 h. Subsequently, the decalcified tissues were dehydrated in an ethanol gradient, embedded in paraffin after dehydration, sectioned, and stained. To evaluate the inflammation induced by infection, H&E staining was performed.

**1.8. *In vivo* bone repairing ability evaluation:** The rats were euthanized 4 and 8 weeks after implantation, and the femurs were harvested and fixed with 4% paraformaldehyde for 24 hours at room temperature. Then, a micro-computed tomography scan was performed for evaluating new bone formation.

**1.9. Micro-CT analysis.** To investigate the healing status of bone defects, rats from each group were euthanized at 4- and 8-weeks post-implantation, and the femurs were harvested and fixed in 4% paraformaldehyde at room temperature for 24 h. Subsequently, Micro-CT scanning was performed to assess new bone formation. For quantitative comparison, parameters including bone volume/total volume (BV/TV), trabecular thickness (Tb. Th), trabecular number (Tb. N), and trabecular separation (Tb. Sp) were measured.

**1.10.** **Histological Analysis.** The harvested femurs were promptly fixed in 4% paraformaldehyde, followed by 24 hours of decalcification in JYBL-Ⅰ Decalcifying Solution (Solarbio, China). Subsequently, the tissue was subjected to dehydration using an ethanol gradient. Dehydrated tissues were embedded in paraffin, sectioned, and finally stained. To assess inflammation and bacterial infiltration, H&E, and Giemsa staining were performed. Masson staining was performed to evaluate bone destruction.

**1.11. Statistical Analysis.** All experiments were performed in at least three more duplicates. Results were expressed as the mean ± standard deviation (SD). Statistical analyses were performed using (GraphPad Software, USA). For intergroup comparisons, repeated data were examined based on variance analysis. Statistical significance was determined according to the following *P* values: * < 0.05, ** < 0.01, *** < 0.001, and **** < 0.0001. All graphs were prepared using GraphPad Prism (version 9.4.1, USA) and Original 2018.

**2. Supporting figures**


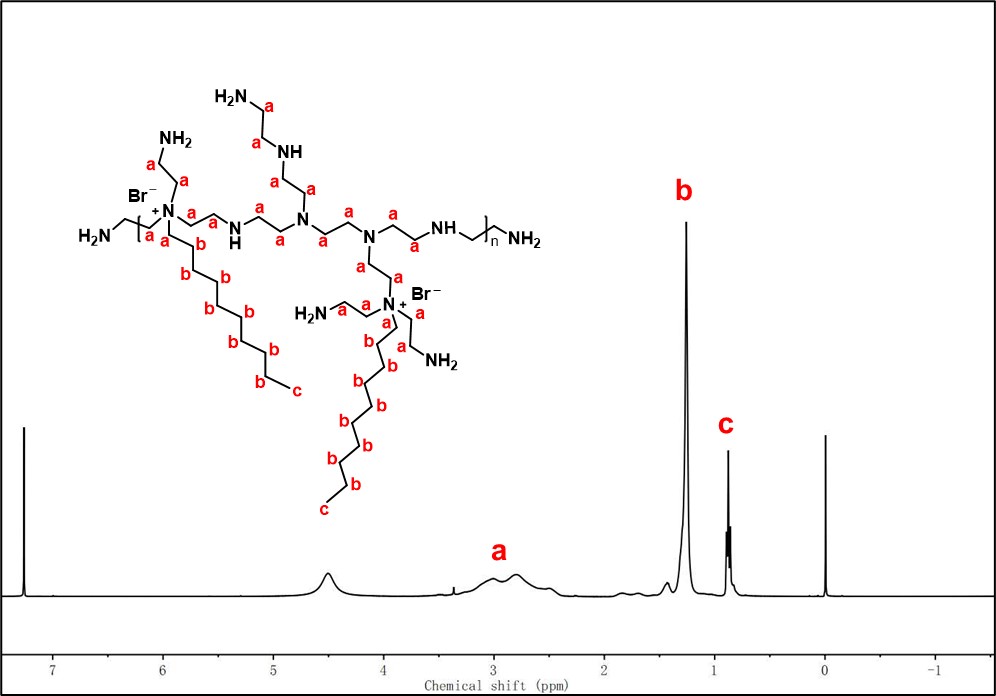


**Fig. S1**. ^1^H NMR spectrum of QPEI.





**Fig. S2**. Thermogravimetric curve of QPEI.


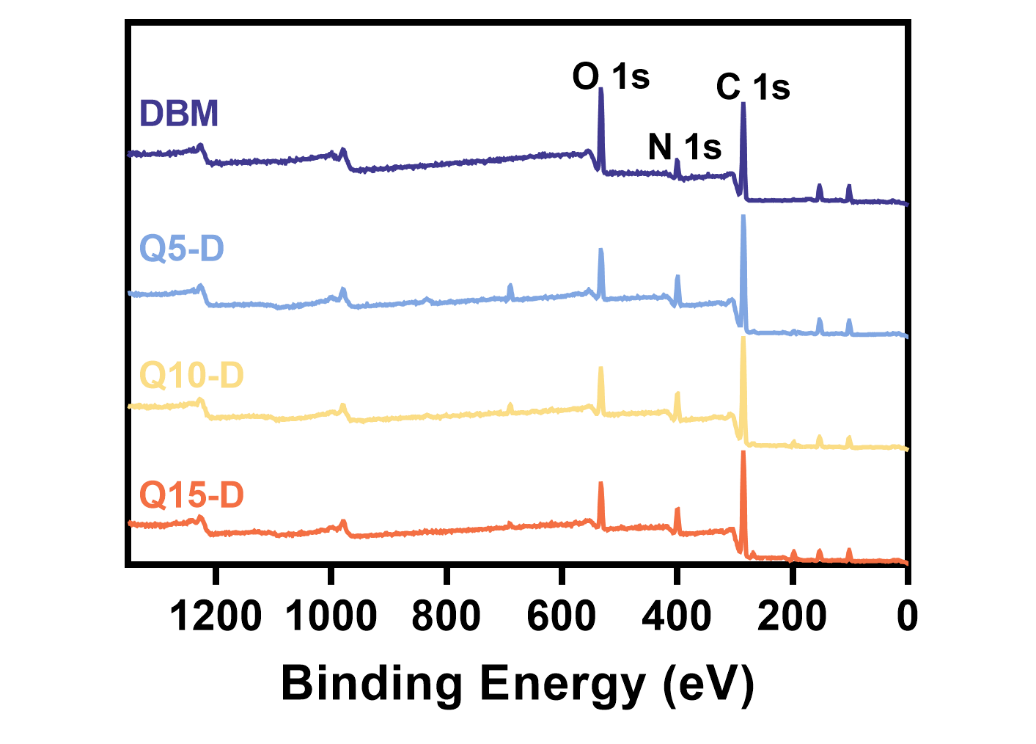


**Fig. S3**. XPS spectra of DBM and Q*x*-D.


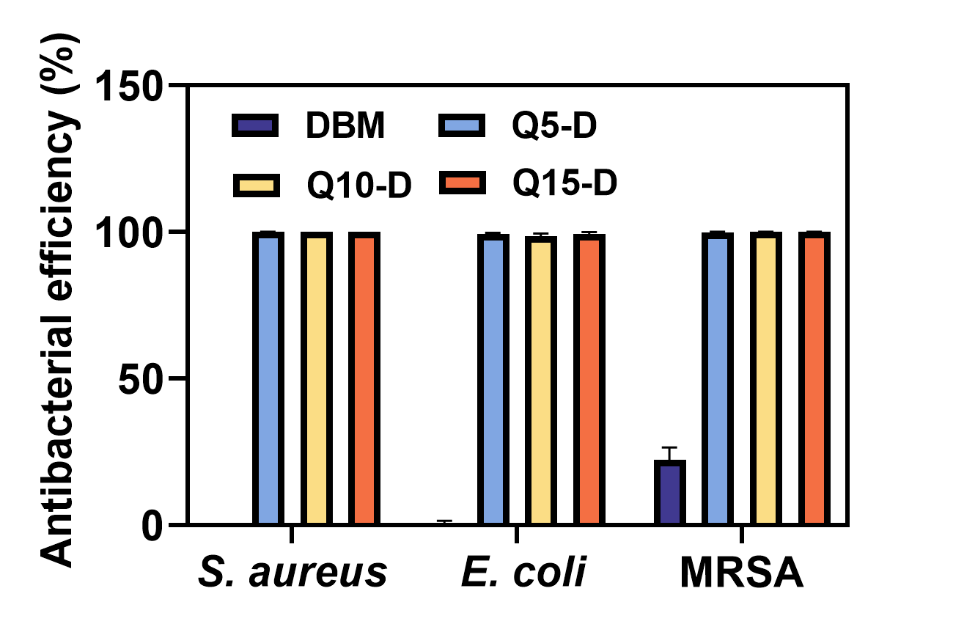


**Fig. S4**. *In vitro* antibacterial efficiency.

**Table S1**. N 1s element ratio of DBM and Q*x*-D

| Sample | N 1s atom % |
| --- | --- |
| DBM | 8.13 |
| Q5-D | 12.7 |
| Q10-D | 12.25 |
| Q15-D | 12.93 |
