# Supplementary figures and images for: Cationized Decalcified Bone Matrix for Infected Bone Defect Treatment
Source: BME Front. 2024 Oct 2;5:0066. doi: 10.34133/bmef.0066 (PMC11445788; doi:10.34133/bmef.0066)

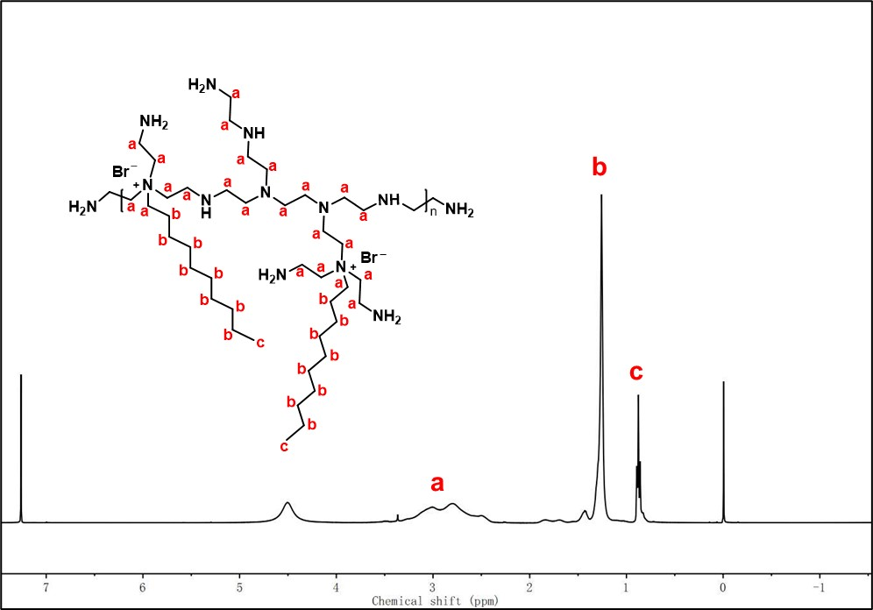

Supplement: Supplementary 1 — Materials and Methods Figs. S1 to S4 Table S1 [file bmef.0066.f1.zip › Figure S1. QPEI_____.tif]

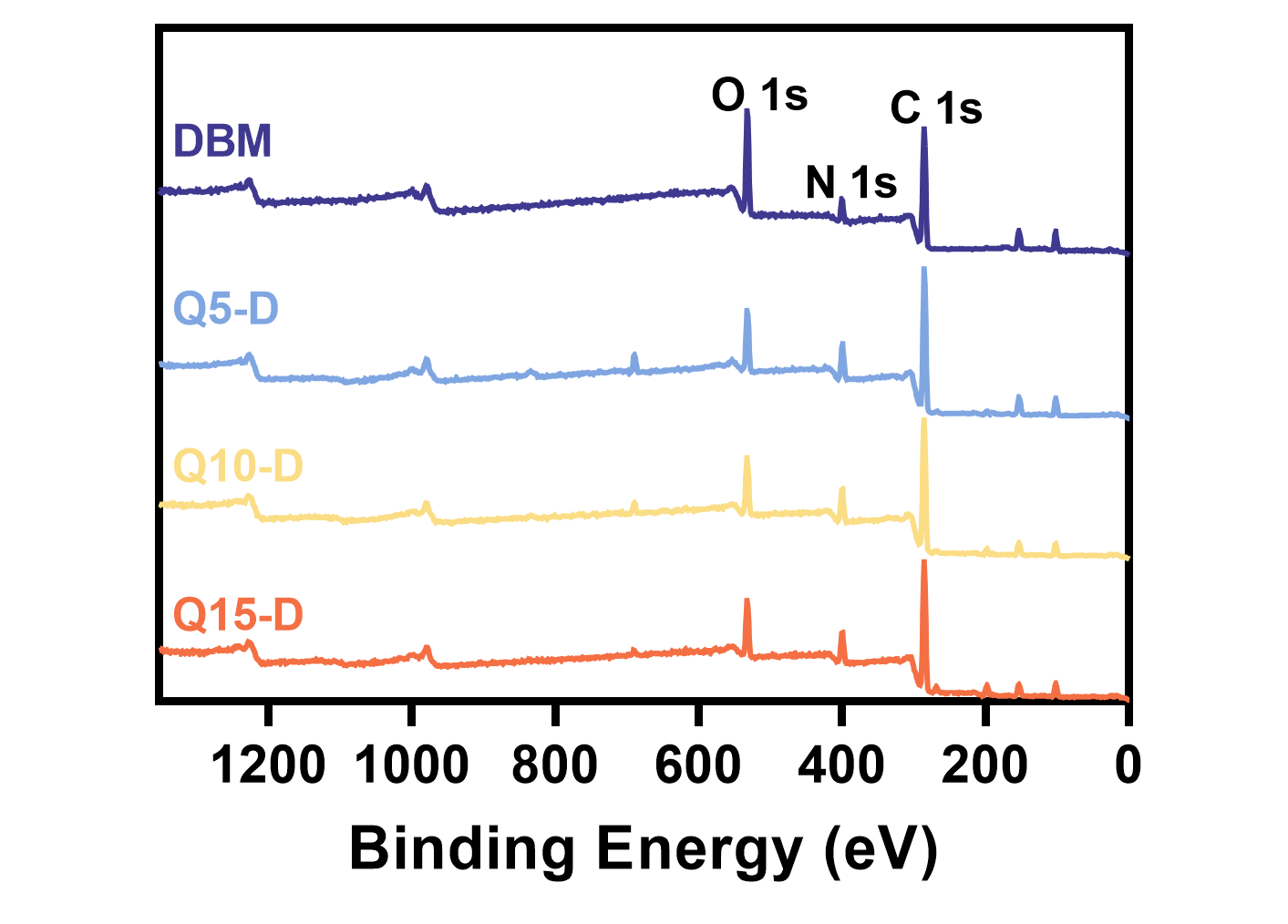

Supplement: Supplementary 1 — Materials and Methods Figs. S1 to S4 Table S1 [file bmef.0066.f1.zip › Figure S3. DBM_Qx-D_XPS__.tif]

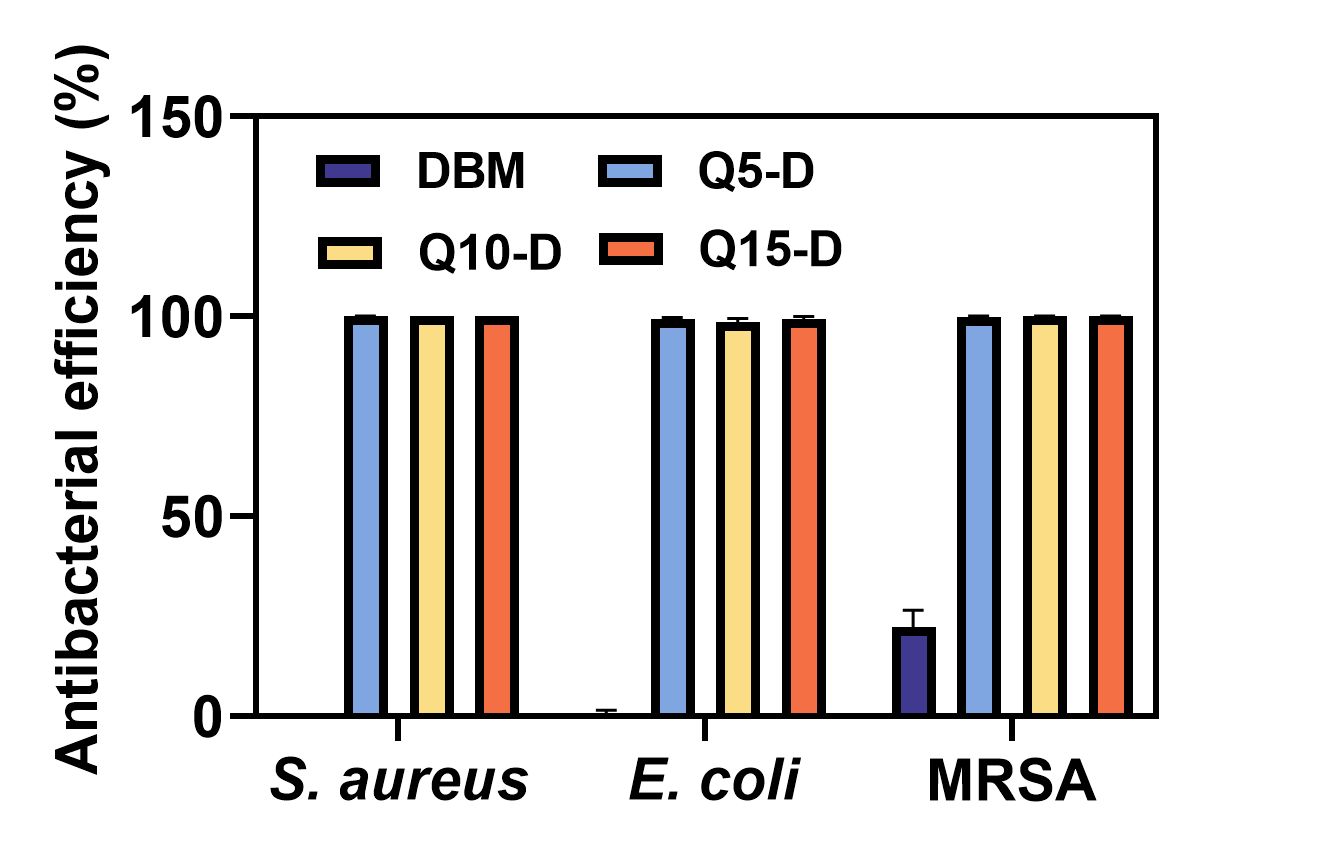

Supplement: Supplementary 1 — Materials and Methods Figs. S1 to S4 Table S1 [file bmef.0066.f1.zip › Figure S4. Qx-D_________.tif]

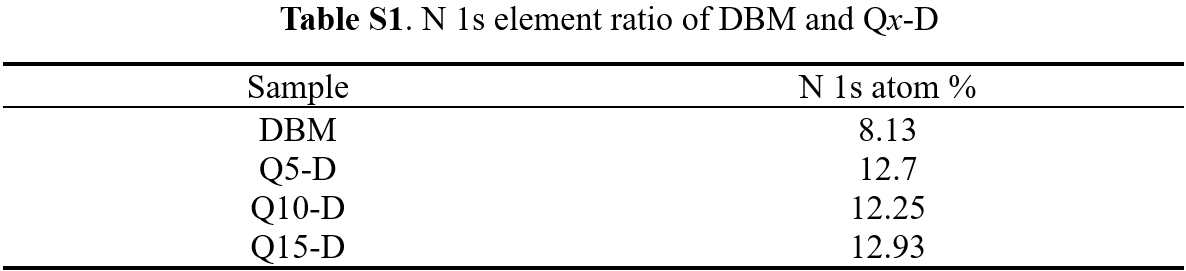

Supplement: Supplementary 1 — Materials and Methods Figs. S1 to S4 Table S1 [file bmef.0066.f1.zip › Table S1. DBM_Qx-D__N1S_____.tif]
